# Cone-Beam Computed Tomographic Assessment of the Mandibular Condylar Volume in Different Skeletal Patterns: A Retrospective Study in Adult Patients

**DOI:** 10.3390/bioengineering9030102

**Published:** 2022-03-02

**Authors:** Chiara Ceratti, Cinzia Maspero, Dario Consonni, Alberto Caprioglio, Stephen Thaddeus Connelly, Francesco Inchingolo, Gianluca Martino Tartaglia

**Affiliations:** 1 Department of Biomedical, Surgical and Dental Sciences, School of Dentistry, University of Milan, 20100 Milan, Italy; cinzia.maspero@unimi.it (C.M.); alberto.caprioglio@unimi.it (A.C.); gianluca.tartaglia@unimi.it (G.M.T.); 2 Fondazione IRCCS Cà Granda, Ospedale Maggiore Policlinico, 20100 Milan, Italy; dario.consonni@unimi.it; 3 Department of Oral and Maxillofacial Surgery, University of California San Francisco, San Francisco, CA 94016, USA; stephen.connelly@ucsf.edu; 4 Interdisciplinary Department of Medicine, University of Bari “Aldo Moro”, 70121 Bari, Italy; francesco.inchingolo@uniba.it

**Keywords:** mandibular condyle volume, cone-beam computed tomography, skeletal pattern, 3D cephalometry

## Abstract

The aim of this study was to assess the condylar volume in adult patients with different skeletal classes and vertical patterns using cone-beam computed tomography (CBCT). CBCT scans of 146 condyles from 73 patients (mean age 30 ± 12 years old; 49 female, 24 male) were selected from the archive of the Department of Dentistry and Maxillofacial Surgery of Fondazione IRCCS Ca’ Granda, Milan, Italy, and retrospectively analyzed. The following inclusion criteria were used: adult patients; CBCT performed with the same protocol (0.4 mm slice thickness, 16 × 22 cm field of view, 20 s scan time); no systemic diseases; and no previous orthodontic treatments. Three-dimensional cephalometric tracings were performed for each patient, the mandibular condyles were segmented and the relevant volumes calculated using Mimics Materialize 20.0^®^ software (Materialise, Leuven, Belgium). Right and left variables were analyzed together using random-intercept linear regression models. No significant association between condylar volumes and skeletal class was found. On the other hand, in relation to vertical patterns, the mean values of the mandibular condyle volumes in hyperdivergent subjects (688 mm^3^) with a post-rotation growth pattern (625 mm^3^) were smaller than in hypodivergent patients (812 mm^3^) with a horizontal growth pattern (900 mm^3^). Patients with an increased divergence angle had smaller condylar volumes than subjects with normal or decreased mandibular plane divergence. This relationship may help the clinician when planning orthodontic treatment.

## 1. Introduction

The mandibular condyle morphology represents an important anatomic region for the skeletal and occlusal relationship as it plays an important role in orthopaedic-orthodontic treatment, both in shape and volume variables [1,2]. The condyle can constantly reshape itself when exposed to various stimuli as it represents a growth site, and the morphology it assumes has a fundamental role in the development and correction of the different types of malocclusions [3,4]. Condyle anatomy should be considered when proposing an orthodontic treatment plan, as a correlation with vertical facial morphology has been hypothesized [3].

The mandibular condyle, as part of the temporomandibular joint (TMJ), is believed to have a key role in the long-term stability of the occlusion after orthodontic and orthognathic treatments.

There are many factors, such as trauma, inflammatory-degenerative diseases or alteration of the position of the mandibular condyle in the temporal fossa, which can alter the balance within the TMJ, resulting in pain, dysfunction, condylar growth problems and morphological or volumetric changes in the condyles [1,5,6].

In orthodontics, the mandibular condyle is typically analyzed through 2D imaging, such as panoramic radiography and lateral cephalogram. The 2D assessment of the TMJ is complex due to its articulated anatomy, magnification errors and the overlapping of adjacent anatomical structures [7,8]. Nowadays, 3D technology allows for a more detailed analysis of the mandibular condyles, their morphology and, above all, their volumes [9].

Cone-beam computed tomography (CBCT) is considered the gold standard for radiographic imaging of the skeletal anatomy of the oral cavity and facial complex [10]. CBCT allows, with a single, low-radiation-dose scan, a complete and reliable 3D analysis with less radiation exposure than the traditional CT scan [7,11]. Three-dimensional, virtual models of the maxillofacial region, including the mandibular condyles, can be rendered from CBCT data sets for accurate diagnosis.

A reliable 3D rendering of condyles using cone-beam computed data was demonstrated in [12,13,14].

The relationship between sagittal and vertical skeletal patterns and mandible condylar volume was also studied. Data on the volume of the mandibular condyle may be indicative and predictive of a precise clinical situation and useful for preventing risk factors for certain TMJ diseases. Evidence showed that the mandibular cortical bone of the condyle head may vary, especially in subjects with different vertical facial patterns [15,16]. The correlation between those variables has not been distributed into orthodontic parameters, such as skeletal classes (I, II and III), and vertical parameters, such as the rotation patterns of the mandible growth, have not been compared with all the variables regarding the healthy patient. Thus, it is extremely important to three-dimensionally understand the correlations and characteristics for orthodontic practitioners.

The aim of this study was to assess the volumes of the mandibular condylar head in a group of adult subjects clinically asymptomatic without any TMJ dysfunction and to verify a correlation hypothesis between these volumes and skeletal features on sagittal and vertical planes using CBCT scans and 3D cephalometry.

## 2. Materials and Methods

This study was made by retrospectively collecting the CBCT scans of patients presented at the Department of Biomedical Surgical and Dental Sciences of the University of Milan, Fondazione IRCCS Ca’ Granda, Ospedale Maggiore Policlinico, Milan. The study protocol was approved by the Ethical Committee of the Fondazione IRCCS Ca’ Granda, Ospedale Maggiore, Milan, Italy. Signed, informed consent for releasing diagnostic records for research purposes was obtained from all the patients included in the study.

A total of 73 patients (49 females and 24 males) were selected according to specific inclusion criteria: Caucasian subjects, all adults aged over 18, healthy, with no systemic disease or genetic syndromes of dental interest; they had not undergone orthodontic treatments or maxillofacial surgery before the examination was performed, and all turned to the Orthodontics Department of the University of Milan for an orthodontic consultation. To assess the absence of TMJ pathologies, the following adjunct parameters were taken into account regarding TMJ range of motion: maximum mouth opening (MMO) within normal ranges (MMO ≥ 40 mm) and no deviation from the midline in the opening movement (deviation from the midline ≤ 2mm) [17].

CBCTs were performed with the same i-CAT^®^ Cone Beam dental imaging system (1910 N. Penn Road, Hatfield, PA, USA) with common head orientation (with the Frankfurt plane parallel to the ground) and according to the same protocol: 0.4 mm slice thickness, 16 × 22 cm FoV (field of view), 20 s scan time and 0.49/0.49/0.5 voxel size. The raw data were then exported, reconstructed and converted into digital imaging and communications in medicine (DICOM3) file format. The DICOM files were then analyzed.

The 73 CBCT scans were retrieved from the records of patients undergoing examination for one of the following: severe Index of Orthodontic Treatment Need (IOTN) grade 4 and 5, dental roots resorption, bone defects, miniscrews prescription and presence of foreign objects.

The tomographic exams were imported in DICOM format to the software Materialize Interactive Medical Image Control System (Mimics^®^) to calculate the 3D cephalometric tracing and the mandibular condylar volumes. The software allows measurements to be performed and volumetric renderings to be obtained.

**Figure 1:**
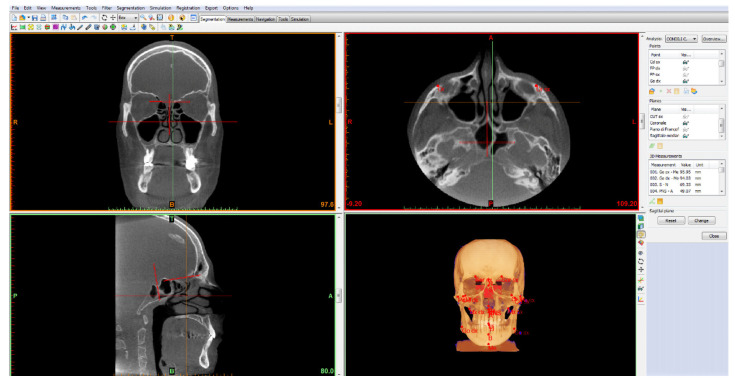
3D cephalometric analysis using the 18-point protocol of the School of Specialization in Orthodontics of the University of Milan, Fondazione IRCCS Ca’ Granda, Ospedale Maggiore Policlinico.

Three-dimensional cephalometry was performed on the CBCT of the patients included in the sample according to the 3D cephalometric analysis proposed by the School of Orthodontics of the University of Milan which identifies 18 landmarks, 10 of which are on the midsagittal plane and 8 of which are lateral and symmetric [8,11] (Figure 1). After identifying the cephalometric points, 36 linear and angular measurements were obtained, all important for achieving information on the position and dimensions of the anatomical structures of orthodontic interest in all three dimensions of space.

Subsequently, the volumes of the mandibular condyles of all the patients examined were calculated. Measurements on the CBCT were performed by three different operators, operator_1_, operator_2_ and operator_3_ (OP1, OP2, OP3).

### 2.1. Mandibular Condylar Segmentation and Volume

**Figure 2:**
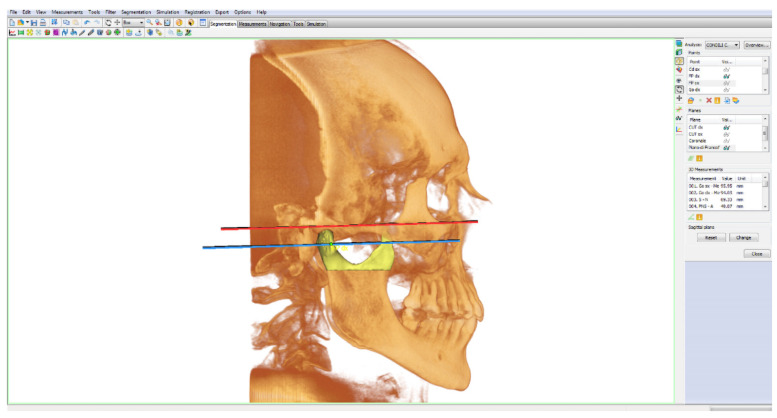
3D rendering highlighting the Frankfurt plane (in red) and the cut plane of the right condyle (in blue) passing through the right pterygoid fovea point (right FP).

The volume of the head of the condyle, which undergoes functional remodeling due to direct contact with the articular disc within the glenoid fossa and on the temporal eminence, was calculated separately [4,13]. As for the calculation of the lower limit of the condylar volume, an anatomical limit was identified: the pterygoid fovea. It is the insertion point of the upper and lower head of the external pterygoid muscle. Its contraction dislocates the condyle and the articular disc forward, downward and inward [18,19]. The cutting plane was chosen parallel to the Frankfurt plane [20], thus, passing through the right and left pterygoid fovea points (Figure 2).

Condyles were isolated before 3D measurements were performed, and the recommended range of bone density (226-3071 HU) was selected.

Three new points and planes to perform the condyle cut were inserted:

Points

- Porion point (right/left Po): highest point of the external acoustic meatus;
- Lower orbital point (right/left OR): lowest point of the orbital edge, the base of the orbital cavity;
- Pterygoid fovea point (right/left FP): most recessed point on the front face of the mandibular neck, identifiable in the three projections: axial, coronal and sagittal. It was chosen as the point that delimits the separation passage between the head and neck of the mandibular condyle.

Planes

- Frankfurt plane: calculated as the plane passing through the lower right and left orbital points (right/left OR) and from the right and left porion points (right/left Po);
- Cut plane of the right condyle (right CUT): plane passing through right FP and parallel to the Frankfurt plane;
- Cut plane of the left condyle (left CUT): plane passing through left FP and parallel to the Frankfurt plane.

**Figure 3:**
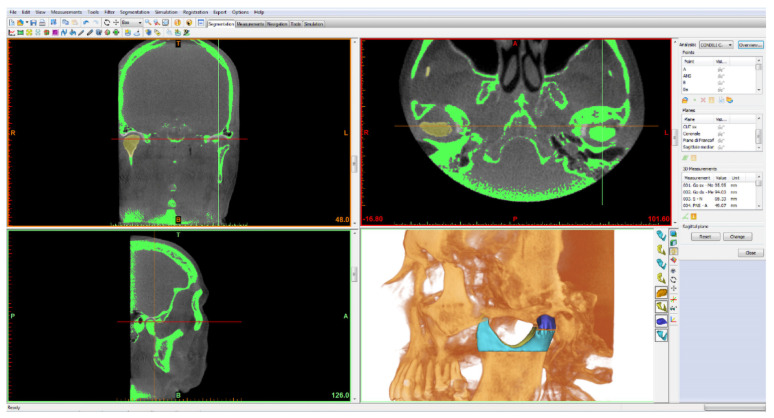
The rendering of the condylar head (blue) and the volume of the condyle portion below (light blue) are visible in the image.

Condyles were segmented by the planes passing through the two right and left “pterygoid fovea” points, parallel to the Frankfurt plane. The volumetric rendering of the head of the condyle was isolated from the neck on both right and left sides. (Figure 3).

The data obtained were exported and organized in a numerical analysis table to carry out the statistical analysis.

### 2.2. Statistics

The overall sample size was calculated with a statistic power of 80% and an alpha 0.05 and delta 0.04 with a probability effect size among the control 0.32 and odds ratio 0.30. The overall resulting sample size was 48 cases; we increased the sample by 20% to match the unavailability of patient data. The variation of condylar volumes in relation to the following parameters was investigated:

Sagittal plane (skeletal class: I, II, III) for values of ANB = 2 ± 2 (°);

Vertical patterns:

- Divergence angle, also called the intermaxillary angle, is the angle formed between the maxillary plane (Ans-Pns) with the mandibular plane (right/left Go-Me). This angle identifies and distinguishes the subjects as: normo/hyper/hypo divergent = 41 ± 1 (°);
- Total goniac angle is the angle that is formed between the body (right/left Go-Me) and the branch of the jaw (right/left Cd-right/left Go) of each side = 120° ± 5°. Increased values of the angle indicate a post-rotation growth pattern while decreased values indicate a horizontal growth.

Sex (female/male).

In the protocol phase, based on preliminary data, we hypothesized a mean condylar volume of 850 mm^3^ in males and 650 mm^3^ in females, with a standard deviation of 250. We intended to analyze at least 20 males and 40 females. With these assumptions, we calculated a power of 82% to detect a significant (*p* < 0.05, two-tailed) mean volume difference between genders.

The effect of side was evaluated, and no differences were found; therefore, left and right variables were analyzed together. To take into account intra-subject correlations, random-intercept linear regression models were fitted to calculate slopes, 95% confidence intervals (CIs) and *p* values.

Crude, adjusted (by sex and age) and stratified (by sex) analyses were performed. Statistical analyses were conducted with Stata 16 (StataCorp., College Station, TX, USA, 2019). To evaluate intra- and inter-operator reliability of the analyzed condylar volumes, three independent observers with the same background performed volumetric analyses of the condyles of three subjects three times each, with an interval of 15 days. ICC estimated intra-rater and inter-rater reliability, and their 95% confident intervals were calculated using S SPSS^®^ 25.00 for Windows (IBM Corporation, Armonk, NY, USA) based on a single-measurement, absolute-agreement, 2-way, mixed-effects model for each variable.

## 3. Results

Mean condylar volume (average of the two condyles) was 141 mm^3^ larger (*p* = 0.02) in males (842 mm^3^, SD 247) than in females (701 mm^3^, SD 233). No important differences were found between right and left condyles in both males (difference 11 mm^3^, *p* = 0.34) and females (difference 27 mm^3^, *p* = 0.22). No interesting association between condylar volumes and skeletal class, I, II or III, was found (Table 1).

Results of clinical interest were obtained in the correlation of condylar volume with vertical patterns: the angle of divergence (Ans–Pns ^ right/left Go−Me) and the total goniac angle (right/left Cd−right/left Go ^ right/left Go−Me) (Table 1). Condylar volumes tended to be larger in hypodivergent and smaller in hyperdivergent subjects (compared to normodivergent) and larger in horizontal growth and smaller in post-rotation growth (compared with normal growth) (Table 1). The average mandibular condylar volume was 141 mm^3^ larger in males (701 ± 241 mm^3^ in females and 842 ± 256 mm^3^ in males).

**Figure 4:**
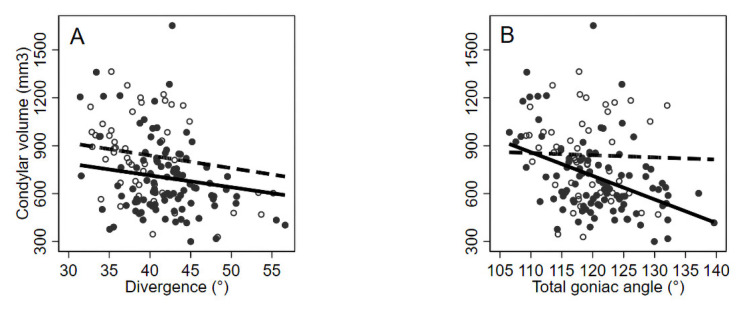
Condylar volume values (mm^3^) in relation to selected continuous variables: divergence (°) (**A**) and Total goniac angle (°) (**B**), by gender. Observed (circles) and crude, predicted, random-intercept linear regression lines. Females: black circles and solid lines; males: empty circles and dashed lines.

The association with divergence was negative and similar in both genders (−7 mm^3^ per degree in females, 95% CI: −17 to +2, *p* = 0.12 and −8 mm^3^ per degree in males, 95% CI: −23 to +7, *p* = 0.29) (Figure 4A). There was a strong negative relationship between volume and total goniac angle in women (−15 mm^3^ per degree, 95% CI: −22 to −8, *p* < 0.001) (Figure 4B). The same analyses adjusted for age confirmed these findings (Table 2).

For the volumetric values calculated, both on the right and left side, intra-rater and inter-rater reliability were very high (ICC > 0.99).

## 4. Discussion

The mandibular condyle plays an important role in orthodontic treatment both in terms of its shape, normally elliptical in adults, and in terms of volume. Bone remodeling starts at the fetal period and continues throughout life. This process occurs through alternating coupled bone apposition and resorption [21], leading to changes in the size and shape of the bony structure [22]. Surfaces of the articular heads are constantly in search of functionally optimal architecture. The mandibular condyle has a unique structure and undergoes constant functional remodeling in response to various mechanical loads that force it to be highly adaptive [23]. In orthodontic treatment, the balance between dentition and related musculoskeletal structures is of great importance. Thus, the condylar position in the glenoid fossa is significant in maintaining or restoring temporomandibular harmony with the dentition and plays an important role in the stability of the occlusion after orthodontic treatment [24,25].

The starting point of this study was the 3D cephalometric analysis, with the protocol proposed by the School of Specialization in Orthodontics of the University of Milan, that allowed us to divide the patients examined in relation to the skeletal variables by identifying 18 points and 36 measurements (Figure 1) using CBCT. It performed synthetic and easily reproducible 3D cephalometric tracing while the risk of undersizing of anatomical elements was lower in comparison with fan-beam computer tomography [26]. As previously mentioned, 3D cephalometric analysis elaborates 3D rendering and 2D planar projections at the same time, keeping the linear and angular quantities close to the reality [8,11].

In this study, only adult patients were selected (over 18 years of age). The choice was made because changes in the condyle volume as a result of occlusal adjustment and functional remodeling during the individuals’ lifetimes were reported [1,23]. The patients with systemic pathologies or genetic syndromes of dental interest and patients who had undergone previous orthodontic treatments or maxillofacial surgeries were excluded.

The volumes of the mandibular condyles were calculated. Regarding condyle segmentation, we proposed a repeatable and precise method for volumetric quantification of the head of the condyle [1,23]. An anatomical point and a known plane were used: the pterygoid fovea point [18,19,25] and the Frankfurt plane [4,15,20]. The condyle segmentation plane was selected parallel to the Frankfurt plane and passing through the right and left “pterygoid fovea” points (Figure 2).

Overall, for volumetric variables, intra-rater and inter-rater reliability were excellent, with ICC > 0.99 (lower limits of 95% confidence intervals all higher than 0.99), thus, showing the adequate performance of data collection and measurement protocols.

The statistical analysis of data allowed us to analyze the results obtained and to compare the condylar volume in relation to skeletal patterns deriving from the 3D cephalometric tracing performed on the CBCT for all patients included in the study.

The analysis of the relationship between condylar volumes and the antero-posterior relative maxillo-mandibular relationships showed how the volumes are distributed among different skeletal classes (Table 1), similar to other previously published studies [27,28,29]. Instead, when the mandibles were categorized according to cephalometric vertical patterns, statistically significant and clinically useful correlations with condylar volume were detected, thus, confirming previous investigations [4,13].

As far as it is known, few clinical studies, using three-dimensional CBCT analysis, evaluated the volume of the mandibular condyle head in adult patients by studying its correlation with vertical cephalometric variables [3,16]. The results of these studies are in line with ours and showed how vertical variables influence condylar remodeling, showing, in particular, how hyperdivergent patients have smaller condyles than hypodivergent patients [3,16,27].

In this study the relationships between the condylar volumes and vertical patterns were statistically significant according to the multiple random-intercept linear regression models.

Both the divergence angle (Ans-Pns ^ right/left Go-Me) and the total goniac angle (right/left Cd-right/left Go ^ right/left Go-Me) were considered (Table 1). Subjects that have increased angle of divergence, i.e., hyperdivergent, have smaller condyles than those with decreased angle of divergence, hypodivergent, which have bigger condylar volumes. Likewise, subjects with increased total goniac angles presenting a post-rotation growth pattern have smaller condyles compared to subjects with a horizontal growth pattern in which the total goniac angle is bigger.

The scatter plot shows how the trend of the volume in relation to divergence was observed to be the same within genders F/M (Figure 4A and Table 2); the only difference being that the condylar volumes of the male subjects were bigger than the condylar volumes of female subjects. Statistically significant higher condylar volumes in male subjects compared to female subjects were found. Results are comparable with those present in literature [4,13,16].

A strong negative association between volume and total goniac angle in women was found (Figure 4B and Table 2). This correlation between vertical patterns and condylar volumes is an interesting fact from a clinical point of view. There are some studies in literature that demonstrated a relationship between muscle activity and vertical skeletal growth [30]. The literature showed that, during mandibular dynamics, there is a functional remodeling response [31]. In particular, the head of the mandibular condyle may react directly or indirectly to the load exerted by the chewing muscles [32]; it varies in thickness and mineralization in relation to different vertical facial models [33,34].

Considering the literature previously reported, it is known that the masticatory muscle activity load is mainly transmitted to the teeth, the surrounding bone and the TMJ [31]. The chewing forces of patients with divergence and increased total goniac angles are closer to the TMJ than in patients with relatively small angles; therefore, the force acting on the condyle of hyperdivergent patients is higher than the force acting on the condyle of hypodivergent patients [35,36]. This leads to the assumption, as reported by this study, that the mandibular condyle might be smaller in patients with increased vertical patterns [3,16].

In addition, other studies also showed that the upper joint space in the glenoid fossa is significantly smaller in the group of hyperdivergent patients and is, therefore, associated with higher-positioned condyles. These studies believed that this trend reflects condylar tissue resorption and predicted a decrease in condylar growth potential. Patients with increased vertical variable values tend to have smaller and higher-positioned condyles than those with the hypodivergent skeletal model [3,16,27,35].

From a clinical point of view, these results led us to hypothesize that there may be a correlation between the predisposition to temporomandibular disorders and condyle volumes in subjects with different mandibular skeletal divergence. Data on the volume of the mandibular condyle may be indicative and predictive of a precise clinical condition and useful in preventing risk factors for some TMJ diseases. This hypothesis needs to be tested by future studies.

Limitations: The present study was performed without the main objective of analyzing correlations between sexes; therefore, the male/female samples were not paired due to randomized selection. Additionally, the numbers of patients with angle class II and/or with hypo- or hyperdivergent facial patterns were larger than the number of class I or normodivergent patterns. This is due to the clinical origin of the database where more subjects with facial alterations are expected.

## 5. Conclusions

The results of this research confirm that condylar volumes vary considerably in relation to vertical patterns in healthy adult patients. Subjects with increased divergence and total goniac angles have smaller condylar volumes than subjects with normal or decreased mandibular plane divergence.

In conclusion, this study allowed us to investigate the three-dimensional nature of the mandibular condyle head in a unique and new way, revealing how attention should be paid to vertical variables when planning orthodontic treatments and paving the way for further studies.

## Figures and Tables

**Table 1:** Condylar volume values (mm^3^) in relation to selected, categorized variables.

Variable	N Condyles	Median	Mean	Min–Max	SD	*p*-Value *	*p*-Value **
Anb 2 ± 2 (°)							
Class I	42	802	824	301–1362	266	Reference	Reference
Class II	60	665	726	329–1653	249	0.10	0.03
Class III	44	633	703	378–1365	239	0.15	0.08
Divergence 41 ± 1 (°)							
Hypodivergent	59	798	812	378–1365	259	0.50	0.51
Normodivergent	29	727	753	347–1222	229	Reference	Reference
Hyperdivergent	58	624	688	301–1653	246	0.16	0.17
Total goniac angle 120 ± 5 (°)							
Horizontal growth	31	926	900	347–1362	252	0.14	0.31
Normal growth	87	679	733	329–1653	238	Reference	Reference
Post rotation growth	28	596	625	301–1184	229	0.03	0.13

* From univariate, random-intercept linear regression models. ** From multiple random-intercept linear regression models adjusted for gender and age.

**Table 2:** Condylar volume values (mm^3^) in relation to selected, continuous variables, by gender.

Females				Males		
Variable	Slope	95% Confidence Interval	*p*-Value	Slope	95% Confidence Interval	*p*-Value
Anb (°)	−1	−17 to +16	0.94	−38	−63 to −14	0.002
Divergence (°)	−7	−15 to +2	0.14	−8	−23 to +7	0.29
Total goniac angle (°)	−10	−17 to −4	0.003	−1	−15 to +12	0.83

From multiple random-intercept linear regression models adjusted for age.

## Data Availability

The data presented in this study are available on request from the corresponding author. The data are not publicly available due to privacy reasons.
